# Use of At-Home COVID-19 Tests — United States, August 23, 2021–March 12, 2022

**DOI:** 10.15585/mmwr.mm7113e1

**Published:** 2022-04-01

**Authors:** Benjamin Rader, Autumn Gertz, A. Danielle Iuliano, Matthew Gilmer, Laura Wronski, Christina M. Astley, Kara Sewalk, Tanner J. Varrelman, Jon Cohen, Rishika Parikh, Heather E. Reese, Carrie Reed, John S. Brownstein

**Affiliations:** ^1^Boston Children’s Hospital, Boston, Massachusetts; ^2^Boston University School of Public Health, Boston, Massachusetts; ^3^CDC COVID-19 Emergency Response Team; ^4^General Dynamics Information Technology, Atlanta, Georgia; ^5^Momentive, San Mateo, California; ^6^Broad Institute of Harvard and MIT, Cambridge, Massachusetts; ^7^Harvard Medical School, Boston, Massachusetts; ^8^Goldbelt C6, Chesapeake, Virginia.

COVID-19 testing provides information regarding exposure and transmission risks, guides preventative measures (e.g., if and when to start and end isolation and quarantine), identifies opportunities for appropriate treatments, and helps assess disease prevalence (*1*). At-home rapid COVID-19 antigen tests (at-home tests) are a convenient and accessible alternative to laboratory-based diagnostic nucleic acid amplification tests (NAATs) for SARS-CoV-2, the virus that causes COVID-19 (*2*–*4*). With the emergence of the SARS-CoV-2 B.1.617.2 (Delta) and B.1.1.529 (Omicron) variants in 2021, demand for at-home tests increased^†^ (*5*). At-home tests are commonly used for school- or employer-mandated testing and for confirmation of SARS-CoV-2 infection in a COVID-19–like illness or following exposure (*6*). Mandated COVID-19 reporting requirements omit at-home tests, and there are no standard processes for test takers or manufacturers to share results with appropriate health officials (*2*). Therefore, with increased COVID-19 at-home test use, laboratory-based reporting systems might increasingly underreport the actual incidence of infection. Data from a cross-sectional, nonprobability–based online survey (August 23, 2021–March 12, 2022) of U.S. adults aged ≥18 years were used to estimate self-reported at-home test use over time, and by demographic characteristics, geography, symptoms/syndromes, and reasons for testing. From the Delta-predominant period (August 23–December 11, 2021) to the Omicron-predominant period (December 19, 2021–March 12, 2022)^§^ (*7*), at-home test use among respondents with self-reported COVID-19–like illness^¶^ more than tripled from 5.7% to 20.1%. The two most commonly reported reasons for testing among persons who used an at-home test were COVID-19 exposure (39.4%) and COVID-19–like symptoms (28.9%). At-home test use differed by race (e.g., self-identified as White [5.9%] versus self-identified as Black [2.8%]), age (adults aged 30–39 years [6.4%] versus adults aged ≥75 years [3.6%]), household income (>$150,000 [9.5%] versus $50,000–$74,999 [4.7%]), education (postgraduate degree [8.4%] versus high school or less [3.5%]), and geography (New England division [9.6%] versus West South Central division [3.7%]). COVID-19 testing, including at-home tests, along with prevention measures, such as quarantine and isolation when warranted, wearing a well-fitted mask when recommended after a positive test or known exposure, and staying up to date with vaccination,** can help reduce the spread of COVID-19. Further, providing reliable and low-cost or free at-home test kits to underserved populations with otherwise limited access to COVID-19 testing could assist with continued prevention efforts.

Information regarding COVID-19 symptoms, testing practices, demographics, and geography were collected from an ongoing, prospective, nonprobability–based cross-sectional online survey^††^ among 418,279 U.S. adults aged ≥18 years during August 23, 2021–March 12, 2022. This previously validated (*8*) COVID-19 survey is a collaboration between the OutbreaksNearMe (a participatory surveillance system) team,^§§^ and Momentive, the developers of the online survey platform SurveyMonkey. Persons were invited at random to participate in the COVID-19 survey following completion of an unrelated survey on the SurveyMonkey platform, which has a diverse user base of approximately 2 million daily respondents^¶¶^ (*8*). Respondents at each unique Internet Protocol address (as a proxy for a unique household) could participate once. Respondents were not compensated or offered incentives. Survey data were weighted for age, race/ethnicity,*** sex, education, and geography^†††^ using the U.S. Census Bureau’s American Community Survey^§§§^ to approximate the demographic composition of U.S. adults (Supplementary Table 1, https://stacks.cdc.gov/view/cdc/115598). Respondents with missing demographic information required to generate weights (e.g., age) (3,435; 0.8%) were excluded from analysis. Persons who reported symptoms^¶¶¶^ during the preceding 7 days were asked if they had been tested for COVID-19 in the preceding 30 days, and, if yes, the type of test used. Starting September 13, 2021, respondents who did not report symptoms were also asked if they had been tested for COVID-19 during the preceding 30 days and, if yes, the type of test used. Respondents could only report a single test and result. Respondents who reported a COVID-19 test were asked the reasons for testing and could select multiple reasons from nine options including “other.” Descriptive analyses of the proportion and associated 95% CI**** of adults reporting at-home test use across self-reported demographic characteristics, geography, and Delta- and Omicron-predominant periods were conducted. Two subgroups were analyzed: 1) those with COVID-19–like illness, to assess symptomatic at-home test use and the symptoms associated with testing, and 2) those who used any diagnostic COVID-19 test, to compare at-home test use with tests administered in other settings. Finally, reasons for using at-home tests and other COVID-19 diagnostic tests were compared. R (version 3.6.2; R Foundation) was used to conduct analyses. This study was approved by the Boston Children’s Hospital Institutional Review Board and received a waiver of informed consent.

Self-reported at-home test use increased during the study period (Figure). At-home test use peaked in January 2022, with 11.0% (95% CI = 10.7%–11.3%) of the surveyed population reporting at-home test use within the preceding 30 days compared with 2.0% (95% CI = 1.8%–2.1%) in October 2021 and 7.5% (95% CI = 7.1%–8.0%) in March 2022. Among persons with COVID-19–like illness, at-home test use increased from an average of 5.7% (95% CI = 5.2%–6.3%) during the Delta-predominant period to 20.1% (95% CI = 19.0%–21.2%) during the Omicron-predominant period.

**FIGURE:**
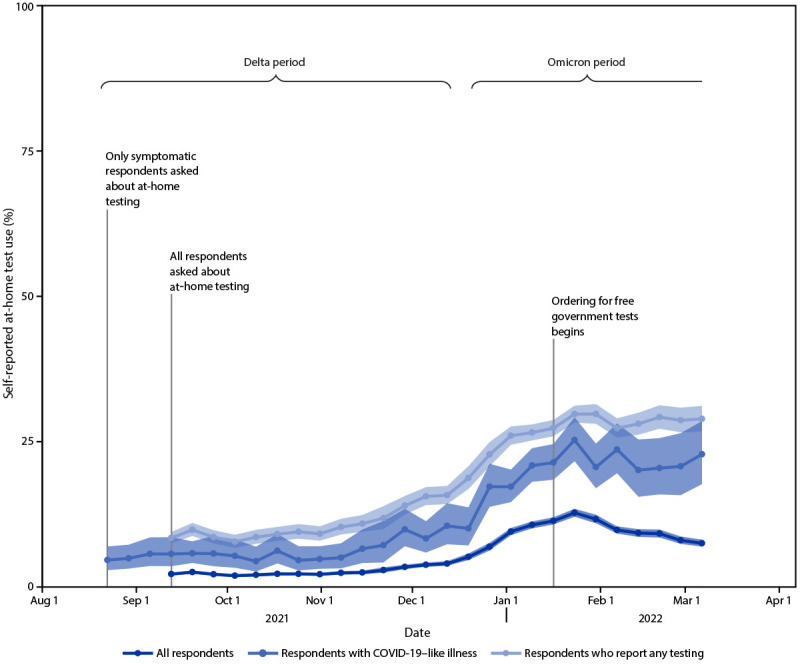
Proportion* of adults aged ≥18 years who reported at-home rapid COVID-19 antigen test use during the preceding 30 days — United States, August 23, 2021–March 12, 2022^†^^,^^§^ * 95% CI indicated by shading. ^†^ B.1.617.2 (Delta)-predominant period = August 23–December 11, 2021; B.1.1.529 (Omicron)-predominant period = December 19, 2021–March 12, 2022 (end of study period). ^§^ Data aggregated by epidemiologic week to reduce noise related to daily estimates. Data points for each week displayed on the first day of respective *MMWR* week.

Persons who identified as White were approximately twice as likely to report at-home test use (5.9%) compared with those who identified as Black (2.8%) (Table 1). Adults aged 30–39 years were more likely to report at-home test use (6.4%) than were those aged 18–29 years (5.1%) and ≥75 years (3.6%). At-home test use also increased with higher levels of household income and education. At-home test use was reported by a higher percentage of persons with annual U.S. household incomes >$150,000 (9.5%) compared with the $50,000–$74,999 (U.S. median household income) range (4.7%), as well as persons with a postgraduate degree (8.4%) compared to person with a high school degree or less (3.5%). By U.S. Census Division, respondents in the New England division reported the highest at-home test use over the study period (9.6%; 95% CI = 9.0%–10.1%), and those in the West South Central division reported the lowest use (3.7%; 95% CI = 3.5%–4.0%) (Supplementary Figure, https://stacks.cdc.gov/view/cdc/115600).

**TABLE 1:** Percentage of survey respondents reporting at-home rapid COVID-19 antigen test use in the preceding 30 days among a cross-section of adults (N = 359,399*) aged ≥18 years, by demographic and other characteristics**^†^** — United States, September 13, 2021–March 12, 2022

Characteristic	Reported at-home test use, % (95% CI)		
	All survey respondents**^§^**	Respondents reporting COVID-19 test^¶^	Respondents reporting COVID-19–like illness symptoms**
**Race** ^††^			
American Indian or Alaska Native	3.3 (2.6–4.1)	10.0 (8.0–12.4)	7.8 (3.9–13.7)
Asian	4.7 (4.3–5.1)	18.3 (16.9–19.8)	15.3 (11.1–20.3)
Black or African American	2.8 (2.6–3.0)	8.8 (8.2– 9.3)	7.6 (5.9– 9.6)
Hispanic or Latino	4.5 (4.2–4.7)	14.1 (13.4–14.9)	13.9 (11.9–16.0)
Native Hawaiian or other Pacific Islander	3.5 (2.5–4.8)	11.2 (8.0–15.2)	8.0 (3.2–16.0)
White	5.9 (5.8–6.1)	22.8 (22.4–23.2)	13.6 (12.9–14.4)
Multiracial	5.4 (4.3–6.7)	17.9 (14.4–21.9)	20.1 (10.6–33.0)
Single other race	4.6 (4.1–5.2)	17.3 (15.5–19.3)	11.8 (8.2–16.2)
**Gender^†^**			
Female	5.4 (5.2–5.5)	19.2 (18.8–19.6)	12.8 (12.1–13.6)
Male	4.9 (4.8–5.1)	18.0 (17.5–18.5)	13.2 (11.9–14.5)
Transgender or nonbinary	6.5 (5.7–7.5)	21.3 (18.7–24.0)	17.7 (13.4–22.6)
**Highest level of education^†^**			
High school or less	3.5 (3.3–3.7)	13.0 (12.4–13.6)	9.7 (8.5–11.0)
Some college	4.8 (4.6–4.9)	17.3 (16.8–17.9)	11.5 (10.5–12.5)
College or more	7.2 (7.0–7.4)	25.7 (25.1–26.4)	18.8 (17.5–20.2)
Postgraduate degree	8.4 (8.1–8.6)	27.8 (27.2–28.5)	20.3 (18.7–21.9)
**Age group, yrs^†^**			
18–29	5.1 (4.9–5.4)	16.9 (16.1–17.7)	13.4 (11.6–15.3)
30–39	6.4 (6.1–6.6)	19.7 (19.0–20.5)	15.3 (13.8–16.9)
40–49	5.8 (5.6–6.0)	19.3 (18.6–20.0)	14.8 (13.4–16.3)
50–64	4.9 (4.8–5.1)	18.8 (18.3–19.3)	11.8 (10.8–12.9)
65–74	4.2 (4.0–4.4)	19.4 (18.5–20.2)	10.0 (8.5–11.7)
≥75	3.6 (3.2–3.9)	17.7 (16.1–19.3)	12.4 (8.9–16.6)
**Annual household income^†^**			
<$15,000	3.1 (2.9–3.4)	10.3 (9.6–11.1)	6.9 (5.6– 8.4)
$15,000–$29,999	3.4 (3.2–3.7)	12.2 (11.4–13.0)	7.2 (5.9– 8.6)
$30,000–$49,999	4.0 (3.8–4.2)	14.9 (14.1–15.7)	11.3 (9.7–12.9)
$50,000–$74,999	4.7 (4.5–5.0)	18.1 (17.3–18.9)	13.1 (11.5–14.9)
$75,000–$99,999	5.6 (5.3–5.8)	20.7 (19.7–21.6)	16.2 (14.2–18.4)
$100,000–$150,000	6.8 (6.5–7.0)	24.7 (23.8–25.6)	20.0 (17.9–22.2)
>$150,000	9.5 (9.2–9.8)	30.0 (29.2–30.9)	25.4 (23.0–27.9)
Did not respond	4.2 (3.9–4.5)	17.5 (16.3–18.8)	12.8 (10.0–16.2)
**COVID-19 vaccination status^†^**			
Unvaccinated	3.5 (3.3–3.7)	13.2 (12.5–13.8)	8.5 (7.3– 9.8)
Partially vaccinated	3.8 (3.5–4.1)	12.9 (12.0–13.9)	11.7 (9.5–14.1)
Fully vaccinated	4.1 (4.0–4.3)	15.7 (15.2–16.1)	11.8 (10.8–12.8)
Fully vaccinated plus booster dose	9.2 (9.0–9.5)	30.0 (29.4–30.6)	21.7 (20.2–23.2)
Did not respond	3.7 (3.0–4.6)	12.1 (9.7–14.9)	8.1 (3.6–15.3)
**Essential worker^†^**			
Yes	5.3 (5.2–5.5)	17.9 (17.5–18.4)	15.8 (14.6–16.9)
No	6.8 (6.6–7.0)	24.0 (23.4–24.7)	17.7 (16.2–19.3)
Did not respond	3.9 (3.8–4.1)	15.8 (15.3–16.4)	8.5 (7.6– 9.4)

* Respondent numbers do not match total for complete survey because the data in this table are restricted to September 13, 2021–March 12, 2022, the time frame when all respondents (not just those who reported being symptomatic) were asked to report their at-home test use.

^†^ The Rao-Scott chi-square test was used to test differences in proportion of respondents that reported having used an at-home test separately, across each of seven categorical variables (i.e., race, gender, age group, income, education, vaccination status, and being an essential worker). Differences were evaluated within each of three subpopulations of interest (i.e., all respondents, those who reported a COVID-19 test, and those who reported COVID19–like illness). All chi-square tests were statistically significant at the Bonferroni corrected p-value threshold of 0.0024 (0.05 over 21 comparisons performed).

^§^ Percentage of respondents who used an at-home test among the entire population of survey respondents, which includes those who used at-home tests, those who used other types of COVID-19 tests, and those who did not test for COVID-19.

^¶^ Percentage of persons who reported at-home test use among the portion of the surveyed population that reported being tested for COVID-19 including those who used at-home tests and those who used other types of COVID-19 tests.

** Percentage of persons who used an at-home test among the portion of the surveyed population that reported symptoms that were consistent with COVID-19–like illness.

^††^ Persons self-identified race/ethnicity based on a list that included U.S. Census Bureau categories for race and Hispanic ethnicity. Persons who selected multiple categories were considered multiracial. Persons who did not select Hispanic were assumed to be non-Hispanic.

Among the surveyed population, the most common reported reasons for at-home test use were for risk assessments, such as COVID-19 exposure concerns (39.4%) and experiencing self-assessed COVID-19 symptoms (28.9%) (Table 2). Risk assessment was reported more often than were logistical or mandated testing reasons (e.g., required for work or school [10.6%] and before traveling [9.2%]). Among persons who were symptomatic, at-home test use was more likely among those whose symptoms were consistent with influenza-like illness (17.0%) than among those whose symptoms were consistent with the COVID-19–like illness case definition (12.2%) (Supplementary Table 2, https://stacks.cdc.gov/view/cdc/115599).

**TABLE 2:** Self-reported reasons for COVID-19 testing among adults aged ≥18 years who reported having received COVID-19 testing in the preceding 30 days, by test type — United States, September 13, 2021–March 12, 2022

Reported reason for testing*	% Reporting (95% CI)	
	Among those using at-home rapid COVID-19 antigen test (n = 18,578^†^)	Among those using other COVID-19 test (n = 80,851^†^)
Exposed to COVID-19	39.4 (38.5–40.3)	19.4 (19.0–19.7)
Had COVID-19 symptoms	28.9 (28.1–29.7)	16.7 (16.3–17.0)
Didn't feel well	28.6 (27.8–29.4)	7.0 (6.7–7.2)
To visit family	17.0 (16.4–17.7)	5.5 (5.3–5.7)
For work/school	10.6 (10.1–11.3)	17.4 (17.0–17.7)
Wanted to travel	9.2 (8.7–9.8)	23.2 (22.8–23.6)
Returning from travel	8.8 (8.3–9.3)	7.8 (7.5–8.0)
Doctor suggested	3.7 (3.4–4.2)	8.4 (8.2–8.7)
Surgery required testing	2.0 (1.7–2.3)	6.4 (6.2–6.6)
Other reported reason	10.3 (9.8–10.9)	13.0 (12.7–13.3)

* Additional response options were added after the question was first implemented on the survey. These response categories were not analyzed because of incomplete data for the study period.

^†^ Eighteen respondents that reported using an at-home test and 86 respondents who reported using other COVID-19 tests did not select any reasons for testing and were excluded from these counts and respective column percentages.

## Discussion

This analysis of data from a nonprobability–based sample of U.S. adults found that during August 23, 2021–March 12, 2022, adults increasingly used at-home tests to evaluate their COVID-19 status. At-home test use especially increased among those with COVID-19–like illness from the period of Delta (5.7%) to Omicron (20.1%) predominance; the latter period coincided with increased availability of at-home test kits and the winter holiday season. As COVID-19 prevalence started to decline in February 2022,^††††^ overall at-home test use also declined. However, among those who reported COVID-19 testing, including those with COVID-19–like illness, the proportion using at-home tests remained stable.

This report found demographic differences in at-home test use. At-home test use was highest among persons who identified as White, adults aged 30–39 years, those with annual household incomes >$150,000, those with postgraduate degrees, and New England division residents. Observed differences might reflect the price point, marketing, education, or disparities in availability and accessibility of at-home tests. Equitable access to COVID-19 testing is important to reduce disease spread. In January 2022, the U.S. government began distributing free at-home tests,^§§§§^ which, if complemented with outreach and communication, might help reduce disparities in COVID-19 testing by alleviating some supply and access barriers (*9*). Additional studies are needed to better understand challenges with testing access, including at-home tests, to develop interventions to reduce barriers and improve access.

With variable access to timely, medically administered tests (e.g., NAATs), coupled with pandemic fatigue, U.S. residents might increasingly rely on at-home tests if such tests are readily available (*2*,*5*). These self-administered at-home tests have a high specificity and moderate sensitivity, which peaks during viral shedding and symptomatic illness. At-home tests can provide valuable information related to community infection incidence and prevalence, even among asymptomatic persons (*2*,*3*). Official COVID-19 surveillance systems aim to capture a comprehensive count of infections. Thus, measuring at-home test use can help quantify the proportion of SARS-CoV-2 infections that might be missed by these systems. These data can also be used to understand reasons for using at-home tests, which were different from those for using tests administered in other settings, and to adapt future surveillance priorities to prevent disease spread. Some manufacturers are providing users with an online process for voluntary reporting of test results to improve tracking of COVID-19 cases. However, implementation of consistent, simple at-home test reporting procedures from all manufacturers could help the continued monitoring of use trends, collect information on new infections, and assist in evaluating interventions (e.g., government test distribution).

The findings in this report are subject to at least seven limitations. First, the survey uses a nonprobability–based sample and the results might not be generalizable to the U.S. population. In addition, information on potential confounders was not collected. For example, information on household size or internet access were neither collected nor adjusted for in weighting. Second, the survey only includes U.S. adults aged ≥18 years. At-home testing patterns among children and adolescents might differ. Third, the survey queried respondents about at-home test use, which was assumed to be at-home rapid antigen tests, although there might be some misclassification given limited but available alternative at-home COVID-19 tests (e.g., concierge and mail-in NAATs). Fourth, the study assessed self-reported at-home test use and did not evaluate the drivers of at-home test use, such as secular trends in accessibility, supply, and ability to locate or afford at-home tests, which might explain observed changes in at-home test use (*10*). Fifth, respondents were asked to report on testing in the preceding 30 days and symptoms in the last 7 days. Those who completed a test in the preceding 30 days but before the appearance of symptoms would be misclassified as testing while symptomatic despite having tested while asymptomatic. Sixth, persons were limited to reporting one test. If persons confirmed the results of an at-home test with a NAAT, they might be more likely to report the latter, more recent one. Finally, respondents were asked to report on past experiences (e.g., testing in preceding 30 days), which might have included periods when a different variant was predominant.

Rapid, at-home diagnostic testing can provide convenient access to assessment of SARS-CoV-2 infection. An increase in U.S. at-home test use from the Delta- to the Omicron-predominant period was observed, with variable use among different demographic groups. Data on at-home test use can provide necessary information to form disease burden estimates. With greater population immunity from vaccines and previous infection, CDC recommends the use of COVID-19 Community Levels to monitor community burden, which include metrics for disease severity and health care system strain.***** Staying up to date with vaccination; testing, including with at-home tests, for persons exposed or with symptoms of COVID-19; appropriate isolation and quarantine; and wearing a well-fitted mask when recommended after a positive test or known exposure are recommended at all COVID-19 Community Levels. Further, providing reliable and low-cost or free at-home test kits to underserved populations with otherwise limited access to COVID-19 testing could assist with continued prevention efforts.

**Summary**

**What is already known about this topic?**

At-home rapid COVID-19 antigen tests (at-home tests) have become widely available in the United States.

**What is added by this report?**

A rapid increase in U.S. at-home test use occurred between the SARS-CoV-2 Delta- and Omicron-predominant periods; at-home test use was lower among persons who self-identified as Black, were aged ≥75 years, had lower incomes, and had a high school level education or less. Commonly reported reasons for using at-home tests included exposure concerns and symptoms.

**What are the implications for public health practice?**

COVID-19 testing, including at-home tests, along with prevention measures such as quarantine and isolation when warranted, wearing a well-fitted mask when recommended after a positive test or known exposure, and staying up to date with vaccination can help reduce the spread of COVID-19. Providing reliable and low-cost or free at-home test kits to underserved populations with otherwise limited access to COVID-19 testing could assist with continued prevention efforts.

## Footnotes

* These authors contributed equally to this report.

†
https://www.whitehouse.gov/briefing-room/press-briefings/2022/01/14/background-press-call-on-the-rollout-of-500-million-free-tests-to-american-homes/

§ Predominance defined as weeks when single variant represented >70% of sequenced specimens. Weeks in which no variant represented >70% of the sequenced specimens were not included in the Delta or Omicron variant periods. Delta-predominant period was defined as August 23–December 11, 2021. The proportions of sequenced viruses that were Delta during the week ending December 4 exceeded 99% and during the week ending December 11 was 92.5%. Omicron-predominant period was defined as December 19, 2021–March 12, 2022. The proportions of sequenced isolates that were Omicron late December 2021 to mid-January 2022 were as follows: week ending December 25 = 71.6%; January 1 = 92.3%; January 8 = 98.3%; and January 15 = 99.5%. Variant co-circulation was observed during the week ending December 18 (Delta = 62.1%, Omicron = 37.6%); therefore, this week was not classified as either Delta- or Omicron-predominant. https://covid.cdc.gov/covid-data-tracker/#variant-proportions

¶ COVID-19–like illness was defined as either cough, shortness of breath, gasping for air, or loss/change of taste/smell or two of the following: fever, chills, aches, headache, sore throat, nausea/vomiting, diarrhea, and fatigue. Syndromic case definitions, including COVID-19–like illness, were applied by researchers posthoc by mapping individually endorsed symptoms to syndromic case definitions. Respondents were included in multiple syndromic categories if their reported signs and symptoms met more than one case definition.

** https://www.cdc.gov/coronavirus/2019-ncov/vaccines/stay-up-to-date.html

†† Full survey is available at https://doi.org/10.6084/m9.figshare.13568648.v4 and the interactive version is available at https://www.surveymonkey.com/r/J8Y7HT7.

§§
https://outbreaksnearme.org/

¶¶
https://www.surveymonkey.com/mp/survey-methodology/

*** Persons self-identified race/ethnicity based on a list that included U.S. Census Bureau categories for race and Hispanic ethnicity. Options included category for other single race. Persons who selected multiple categories were considered multiracial. Persons that did not select Hispanic were assumed to be non-Hispanic.

††† U.S Census Bureau divisions: Division 1 (New England), Division 2 (Middle Atlantic), Division 3 (East North Central), Division 4 (West North Central), Division 5 (South Atlantic), Division 6 (East South Central), Division 7 (West South Central), Division 8 (Mountain), Division 9 (Pacific). https://www2.census.gov/geo/pdfs/maps-data/maps/reference/us_regdiv.pdf

§§§
https://www.census.gov/programs-surveys/acs/data/summary-file.2019.html

¶¶¶ Influenza-like illness was defined as fever in addition to sore throat or cough. Syndromic case definitions, including influenza-like illness, were applied by researchers posthoc by mapping individually endorsed symptoms to syndromic case definitions. Respondents were included in multiple syndromic categories if their reported signs and symptoms met more than one case definition.

****
https://www.cdc.gov/nchs/data/series/sr_02/sr02_175.pdf

††††
https://covid.cdc.gov/covid-data-tracker/#cases

§§§§
https://www.whitehouse.gov/briefing-room/statements-releases/2022/01/14/fact-sheet-the-biden-administration-to-begin-distributing-at-home-rapid-covid-19-tests-to-americans-for-free/

*****
https://www.cdc.gov/coronavirus/2019-ncov/science/community-levels.html
